# The Story of Female Urethral Stricture - “To a man with a hammer, everything looks like a nail”

**DOI:** 10.1590/S1677-5538.IBJU.2021.0292

**Published:** 2021-08-10

**Authors:** Ashwin Shekar, Ganesh Gopalkrishnan

**Affiliations:** 1 Sri Sathya Sai Institute of Higher Medical Sciences Department of Urology Andhra Pradesh India Department of Urology, Sri Sathya Sai Institute of Higher Medical Sciences, Prashantigram, Puttaparthi, Andhra Pradesh, India.

*To the editor*,

In their recent article, Kalra et al. describe the triad to characterize the variable clinical presentation of female urethral stricture (FUS) disease, the diagnostic utility of calibration, video urodynamic study (VUDS), and urethroscopy in planning surgical management (1). Based on their results, they have advocated urethroplasty as an effective surgical solution for even patients who had successful calibration with a 14Fr Foley catheter, with the diagnosis of FUS being based on presence of “stigmata” of stricture disease and video urodynamics (VUDS) findings of bladder outlet obstruction (BOO).

According to literature, FUS are exceedingly rare, with a prevalence of 3-8% overall, 4-13% in women with BOO and 0.1-1% in women with lower urinary tract voiding symptoms (2). Though, of late there has been a plethora of studies focusing on the surgical reconstructive aspect of FUS, unfortunately there is no uniform standard definition of FUS in these studies, with every author describing his own definition (3–5).

In one of the first systematic reviews on this topic, Osman et al. noted that no standardized definition or diagnostic criteria exist for urethral stricture in women. In their review, they failure to admit a 14F catheter was used as an inclusion criterion in only four studies with other studies using urethral caliber thresholds <17F, <19F and <20F. Sixteen studies had included a radiologic evaluation of urethral structuring and the most common definition used was “distal urethral stenosis with proximal urethral ballooning”. The majority of studies in their review reported performing a multitude of these tests, and no authors relied on just one investigative modality (4, 5). In a more recent review by Mmonu et al., FUS were defined as a “fixed”, symptomatic, anatomical narrowing of the urethra that does not accommodate urethral instrumentation (2).

In this paper, the authors have put forth a radical new concept that even in the absence of an anatomical narrowing (successful passage of 17Fr cystoscope), presence of stigmata of stricture disease along with video urodynamic evidence of BOO are enough to diagnose FUS. This paper, though interesting, has some methodological flaws that we would like to highlight. Firstly, the stigmata of stricture that the authors have mentioned has no special significance in these patients, as nearly 75% of the women in the series have undergone repeated dilatations before urethroplasty and hence these changes are expected.

Secondly and more importantly, the VUDS criteria that the authors have used to exclude dysfunctional voiding (DV) is a gross oversimplification, as DV is not just increased sphincter activity during voiding but also can represent a poor relaxation of the sphincter during voiding and this is actually evident in the two VUDS tracings that the authors have shown (6–8). DV/functional BOO is a complex often misunderstood condition, about which, our knowledge is still incomplete and we have reason to believe that many of these patients who have undergone urethroplasty might have been misdiagnosed cases of DV. Though FUS has been an underdiagnosed entity treated with repeated dilatations alone previously, now that we have a better treatment modality in the form of substitution urethroplasty, there is also real possibility of overdiagnosis of this condition. Hence, as clinicians, it is critical that we practice utmost diligence in first diagnosing the condition correctly, before exercising a surgical option.

Another point of concern that we would like to highlight is the short follow-up period in this study, which unfortunately has been the bane of many studies on stricture disease (9). Patients with true FUS also have good short-term outcomes with dilatation, so whether urethroplasty did actually benefit these patients in the form of avoidance of further dilatation can only be commented upon if they had longer follow-up (10). Further, it has to be noted that patients with DV also show good short-term response to dilatation, so the “good” short-term outcomes of urethroplasty cannot be considered as prima facie evidence of a correct diagnosis of stricture in the first place (11).

Nevertheless, the authors need to be congratulated for highlighting this perplexing situation of successful calibration in females diagnosed with BOO, which many urologists face in other everyday practice. We hope papers like these will stimulate further discussion on this unique clinical scenario and in the future will also focus on diagnosis and long-term outcomes of this condition than the specific technique being used.

The Authors
